# A comprehensive resource of genomic, epigenomic and transcriptomic sequencing data for the black truffle *Tuber melanosporum*

**DOI:** 10.1186/2047-217X-3-25

**Published:** 2014-10-30

**Authors:** Pao-Yang Chen, Barbara Montanini, Wen-Wei Liao, Marco Morselli, Artur Jaroszewicz, David Lopez, Simone Ottonello, Matteo Pellegrini

**Affiliations:** 1 Institute of Plant and Microbial Biology, Academia Sinica, Taipei, 11529, Taiwan; 2 Department of Molecular, Cell, and Developmental Biology, University of California, Los Angeles, CA 90095, USA; 3 Laboratory of Functional Genomics and Protein Engineering, Biochemistry and Molecular Biology Unit, Department of Life Sciences, University of Parma, Parma 43124, Italy

**Keywords:** DNA methylation, *Tuber melanosporum*, Ascomycete truffle, Pezizomycetes, Transposable elements, Whole-genome bisulfite sequencing, Methylome, Copy number variation, Transposon expression, 5-azacytidine, Genome plasticity

## Abstract

**Background:**

*Tuber melanosporum*, also known in the gastronomic community as “truffle”, features one of the largest fungal genomes (125 Mb) with an exceptionally high transposable element (TE) and repetitive DNA content (>58%). The main purpose of DNA methylation in fungi is TE silencing. As obligate outcrossing organisms, truffles are bound to a sexual mode of propagation, which together with TEs is thought to represent a major force driving the evolution of DNA methylation. Thus, it was of interest to examine if and how *T. melanosporum* exploits DNA methylation to maintain genome integrity.

**Findings:**

We performed whole-genome DNA bisulfite sequencing and mRNA sequencing on different developmental stages of *T. melanosporum*; namely, fruitbody (“truffle”), free-living mycelium and ectomycorrhiza. The data revealed a high rate of cytosine methylation (>44%), selectively targeting TEs rather than genes with a strong preference for CpG sites. Whole genome DNA sequencing uncovered multiple TE-enriched, copy number variant regions bearing a significant fraction of hypomethylated and expressed TEs, almost exclusively in free-living mycelium propagated *in vitro*. Treatment of mycelia with 5-azacytidine partially reduced DNA methylation and increased TE transcription. Our transcriptome assembly also resulted in the identification of a set of novel transcripts from 614 genes.

**Conclusions:**

The datasets presented here provide valuable and comprehensive (epi)genomic information that can be of interest for evolutionary genomics studies of multicellular (filamentous) fungi, in particular Ascomycetes belonging to the subphylum, Pezizomycotina. Evidence derived from comparative methylome and transcriptome analyses indicates that a non-exhaustive and partly reversible methylation process operates in truffles.

## Data Description

### Purpose of data acquisition

Genome-wide profiles of DNA methylation have recently emerged from methylome studies carried out on more than 20 eukaryotic organisms belonging to four different lineages [1-5]. Promoter and gene-body methylation marks are common in higher eukaryotes where they provide an additional level of gene regulation, while inactivation of transposon and other repeated elements appears to be the main purpose of DNA methylation in fungi (reviewed by [6]).

The black truffle (*T. melanosporum*) is a macrofungus and a highly appreciated gastronomic delicacy produced by an ectomycorrhizal ascomycetous symbiont found throughout southern Europe. It features one the largest genomes (125 Mb), with the highest transposable element (TE) and repetitive DNA content (>58%), amongst fungi that have been sequenced so far [7]. As obligate outcrossing organisms, truffles are bound to a sexual mode of propagation, which together with TEs, has been proposed to be a major force driving the evolution of DNA methylation [6,8]. *T. melanosporum* belongs to the Pezizales, a largely unexplored group of ascomycetes that includes *Ascobolus immersus*, a fungus relying on premeiotic DNA methylation as a means to control repetitive element proliferation [9,10].

In order to investigate how an extremely TE-rich organism, such as *T. melanosporum* exploits DNA methylation to cope with the more than 45,000 repeated elements that populate its genome [11], we have determined the genome-wide bisulfite sequencing (BS-seq) and RNA sequence (RNA-seq) profiles of the saprobic free-living mycelium (FLM), truffle fruitbody (FB) and ectomycorrhizal (ECM) developmental stages of this fungus (see Table 1 for a list of sequencing data and their descriptive statistics). To gain insight on the reversibility of DNA methylation and its effects on TE transcriptional activity, we determined and compared the BS-seq and RNA-seq profiles of untreated FLM and FLM treated with 5-azacytidine (5-aza), a DNA methylation inhibitor. Building on the above data, here we report the results of an in-depth investigation of the relationship between DNA methylation and transcription in genes and TEs, which led to the identification of novel transcripts from individual sample assemblies, as well as from the combined assembly. We also confirmed the relationship between copy number variation (CNV) and DNA methylation by whole-genome sequencing (WGS) of untreated genomic DNA. In this data note we describe how the samples were collected, processed for BS-seq and RNA-seq library construction and analysis, as well as subsequent data mining. We also provide an implementation of the Composite Repeat Induced Point mutation index (CRI), a dinucleotide frequency distribution analysis tool for evaluating the likelihood that DNA methylation in a given genome is induced by “Repeat-Induced Point mutation” (RIP) [12] (see “Availability and requirements of Software Used” for details).

**Table 1:** Summary of sequencing data

**Sample**	**Library**	**Number of raw reads**	**Number of uniquely mapped**	**Mapability**	**Read length**	**Coverage per strand (X)**
**FLM**	BS-seq	122,903,319	78,983,505	64.26%	100	31.61
**FB**	BS-seq	184,692,678	86,778,157	46.99%	100	34.73
**ECM**	BS-seq	182,286,685	3,268,721	1.79%	90	1.18
**5-aza treated**	BS-seq	99,836,913	41,349,427	70.48%	51	8.44
**5-aza untreated**	BS-seq	84,315,198	35,348,536	70.19%	51	7.21
**FLM**	WG-seq	57,386,669	45,239,191	78.83%	51	9.23
**FB**	WG-seq	70,844,918	50,646,551	71.49%	51	10.34
**FLM**	RNA-seq	68,287,049	63,751,666	93.36%	50	N/A
**FB**	RNA-seq	87,310,639	74,090,987	84.86%	50	N/A
**5-aza treated**	RNA-seq	72,050,137	60,644,746	84.17%	51	N/A
**5-aza untreated**	RNA-seq	75,043,437	62,107,389	82.76%	51	N/A

ECM: ECtoMycorrhiza; FB: FruitBody; FLM: Free-Living Mycelium.

### Biological material

*T. melanosporum* (Vittad.) mycelium from the Mel28 strain, the same strain utilized for reference genome sequencing [7], was grown on 1% malt agar (Cristomalt-D, Difal, Villefranche-sur-Saône, France) for 5 weeks before harvesting. A field-collected mature fruitbody was used for the FB library. ECM tips were from common hazel (*Corylus avellana* L.) plantlets inoculated with a *T. melanosporum* mycelium slurry (Raggi Vivai, Cesena, Italy*).* For 5-aza treatment, *T. melanosporum* mycelia were grown in the dark at 23 °C in synthetic liquid medium as described [13]. Every 5 days 5-aza was added to mycelia at 10, 40, and 100 μM final concentrations (from a 10 mM stock solution in water) for 45 days (‘5-aza treated’), with the last addition made 24 hours before harvesting of mycelia and DNA/RNA extraction. The same volume of water, instead of 5-aza, was added to parallel control samples (‘5-aza untreated’).

### Whole-genome bisulfite sequencing

Genomic DNA (gDNA) was extracted by grinding fruitbodies in liquid nitrogen followed by purification with the DNeasy Plant Mini kit (QIAGEN, Hilden, Germany). This was followed by extraction in 50% Phenol-50% extraction buffer (100 mM Tris–HCl pH 8.0, 100 mM NaCl, 20 mM EDTA, 1% SDS) at 65 °C for 10 min and centrifugation at 14,000 rpm for 10 min. The aqueous phase was transferred to new tubes and extracted twice with phenol:chloroform (1:1) and once with chloroform. Following ethanol precipitation, samples were resuspended in H_2_O. LiCl (2 M) precipitation was used to separate RNA (pellet) from gDNA (supernatant). gDNA was precipitated with ethanol and resuspended in H_2_O. Extracted gDNA was further purified with an additional phenol:chloroform extraction and sheared by sonication to generate DNA fragments in the 150–300 bp size range. Bisulfite treatment and library preparation were carried out as described [14], except that the EpiTect kit (QIAGEN) was utilized for bisulfite treatment. Two consecutive rounds of conversion were performed for a total of 10 hours. The resulting libraries were sequenced by Illumina sequencing technology (Hiseq 2000 sequencer; Illumina, San Diego, CA, USA).

### Processing bisulfite converted reads

Bisulfite-converted reads were aligned to the reference genome (Tuber_melanosporum_v1.0) using BS Seeker 2 [15]. We achieved 32X, 35X and 1.2X coverage per strand for FB, FLM and ECM, respectively (see Table 1). The ECM sample contained substantial amounts of root cells from the hazelnut host tree (*C. avellana*), whose genome has not yet been determined. Even at this low coverage, we were able to delineate global, low-resolution methylation profiles for the symbiotic (ECM) stage. Genome-wide DNA methylation profiles were generated by determining methylation levels for each cytosine in the genome. Since bisulfite treatment coupled to PCR amplification converts unmethylated cytosines (Cs) to thymines (Ts), the methylation level at each cytosine was estimated as #C/(#C + #T), where #C is the number of methylated reads and #T is the number of unmethylated reads. The methylation level per cytosine serves as an estimate of the percentage of cells bearing a methylated cytosine at a specific locus.

For subsequent analysis we only considered cytosines that are covered by at least four reads (except for the ECM methylome, which has a considerably lower overall coverage). Table 2 shows that approximately 90% of the cytosines in FB and FLM were retained for analysis and that this percentage drops to approximately 75% for 5-aza treated and untreated. These values suggest our methylation profiles provide a good representation of the truffle methylomes (see Table 3 for the DNA methylation levels of genome, genes and transposable elements in individual BS-seq libraries).

**Table 2:** Number of cytosines included in the methylation analysis

Number of cytosines* (% genome coverage)	**FLM**	**FB**	**ECM**	**5-aza treated**	**5-aza untreated**
**CG**	7,682,955 (92.58%)	7,444,625 (89.71%)	55,380 (0.67%)	6,594,758 (79.47%)	6,147,635 (74.08%)
**CHG**	9,111,984 (93.50%)	8,829,674 (90.61%)	52,152 (0.54%)	7,739,358 (79.42%)	7,054,610 (72.39%)
**CHH**	34,258,661 (91.68%)	32,954,550 (88.19%)	152,076 (0.41%)	28,932,890 (77.43%)	25,963,097 (69.48%)

ECM: ECtoMycorrhiza; FB: FruitBody; FLM: Free-Living Mycelium.

*cytosines with reads coverage > = 4.

**Table 3:** Average methylation levels of genome, genes, and transposable elements

Methylation level*		**FLM**	**FB**	**ECM**	**5-aza treated**	**5-aza untreated**
**Genome**	**CG**	30.3%	28.9%	6.4%	26.4%	25.3%
	**CHG**	8.1%	8.9%	3.4%	7.3%	8.3%
	**CHH**	10.1%	10.3%	3.3%	8.8%	9.9%
**Gene**	**CG**	0.64%	0.87%	0.84%	0.68%	0.68%
	**CHG**	0.36%	0.61%	0.82%	0.39%	0.41%
	**CHH**	0.39%	0.63%	0.95%	0.43%	0.44%
**TE**	**CG**	70.51%	69.71%	59.16%	72.87%	72.89%
	**CHG**	14.85%	16.50%	15.31%	13.99%	16.97%
	**CHH**	17.27%	17.85%	17.30%	16.11%	19.24%

ECM: ECtoMycorrhiza; FB: FruitBody; FLM: Free-Living Mycelium.

*The methylation level is estimated based on all cytosines with reads coverage > = 4 in each sample.

BS-seq data coverage (i.e., read depth) across the genome revealed CNVs between FLM and FB. Specifically, we identified 107 genomic regions with significant CNV (defined as a 100 kb window with a |Log coverage ratio (FLM/FB)| ≥0.3), corresponding to 7.3% of the genome. One hundred and two (95%) of these regions were independently confirmed by standard Illumina sequencing performed on non-bisulfite-treated FLM genomic DNA. This high coincidence rate supports the notion that BS-seq data are unbiased for CNV calling.

### RNA-seq library preparation and data analysis

Total RNA was extracted as described above, dissolved in H_2_O after LiCl precipitation and purified with the RNeasy Plant Mini kit (QIAGEN) followed by on-column DNase I digestion as per manufacturer’s instructions. RNA integrity was verified with a Bioanalyzer (Agilent Technologies, Santa Clara, CA, USA), which yielded RNA integrity number (RIN) scores of 7.0 and 6.5 for FB and FLM, respectively. RNA was quantified with the Qubit RNA BR Assay kit (Life technologies, Carlsbad, CA, USA) and 1 μg of purified RNA was utilized as starting material for library construction, which was carried out with the Illumina TruSeq RNA Sample Preparation kit as per manufacturer's instructions. Libraries were sequenced with an Illumina HiSeq 2000 system using 50-bp single-end reads.

**Figure 1:**
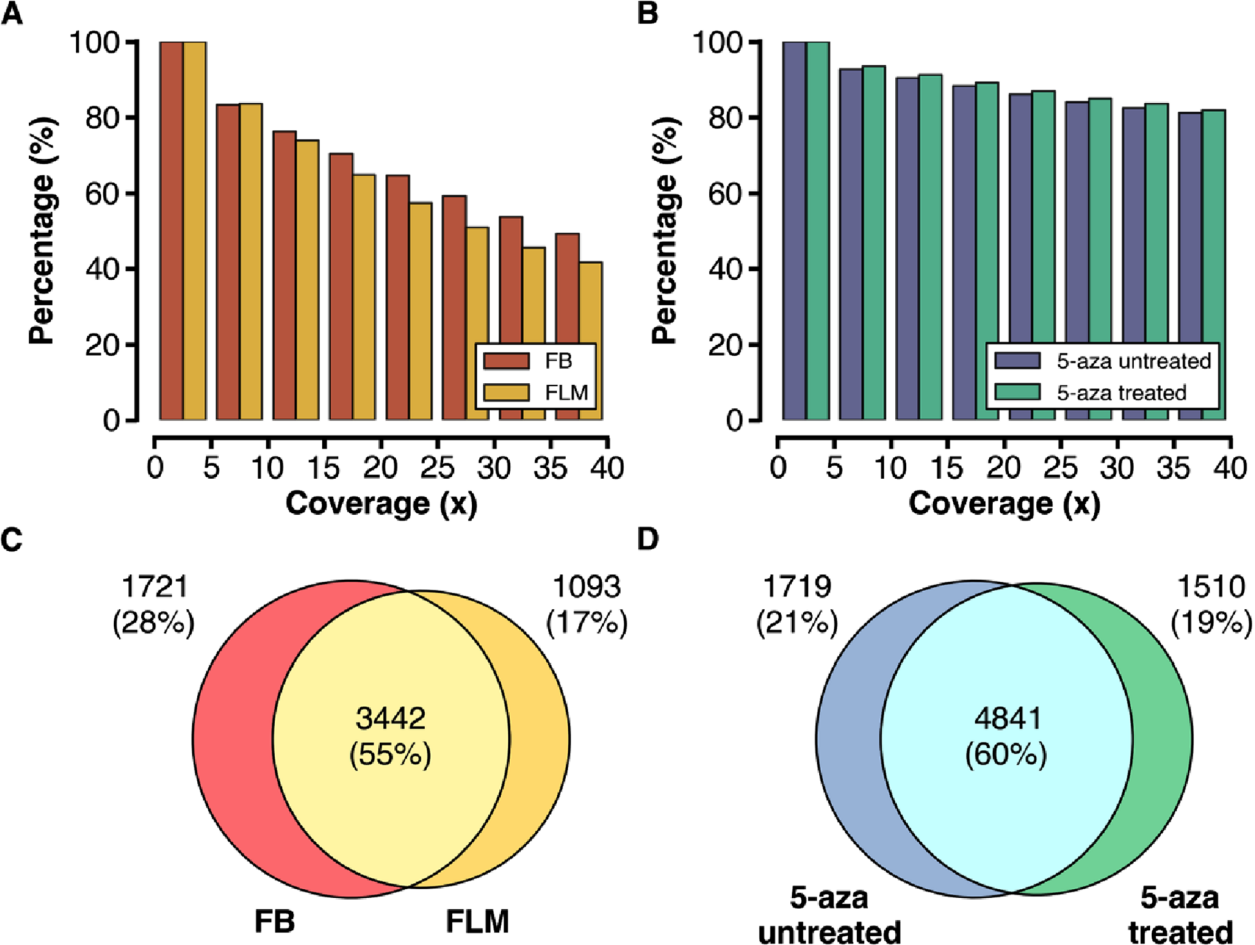
**Coverage of RNA-seq libraries in genes and transposable elements. A**: histogram of read coverage in genes between FB and FLM libraries, and **B**: between 5-aza untreated and treated libraries. **C**: Venn diagram of read coverage in TE between FB and FLM libraries, and **D**: between 5-aza untreated and treated libraries. FB: FruitBody; FLM: Free-Living Mycelium.

The reads were mapped against transcripts of genes and TEs using Tophat [16] allowing up to two mismatches, and only unique alignments were kept. The quality of alignments was checked using FastQC [17]. The resulting alignment files were processed with the HTSeq program (version 0.5.4p3, [18]) to create a gene matrix for downstream analysis. Only reads mapped entirely to a single gene or TE were kept for further analysis. In our FB and FLM datasets, approximately 75% of genes are covered with a depth of at least 10 reads (see Figure 1A). In another set of data comparing 5-aza treated versus untreated FLM samples, about 90% of genes are covered by 10 or more reads (see Figure 1B), suggesting a satisfactory completeness of our data coverage. Reads corresponding to similar, but not identical repeats were also mapped, albeit with a lower ability to be mapped compared to a regular (i.e., distinct sequence element) alignment. The Venn diagrams in Figures 1C and 1D show that 55-60% of TEs are covered in both samples in the two comparisons. In total, 63,742,213 and 74,109,065 reads from FB and FLM, respectively, were uniquely mapped, corresponding to an overall 93.34% and 84.88% mapping. Expression levels were computed as Reads Per Kilobase of exon per Million reads mapped (RPKM) [19]. Gene length was calculated as the union length of all possible exons.

## Methods

### Prediction of novel genes

We used the RNA-seq datasets listed in Table 1 (FB, FLM, FLM treated/untreated with 5-aza) to predict novel genes not included in the original annotation [20]. The prediction pipeline was based on a previously published approach [21]. First, reads were aligned to the reference genome (truffle v1.0 assembly) using TopHat [16], which resulted in the mapping of non-annotated regions. A guided transcript assembly using Cufflinks [22] with the truffle v1.0 assembly was then carried out for each sample. The resulting individual transcript assemblies were compared with the reference assembly using Cuffcompare [22] to identify novel transcripts.

A transcript is deemed novel if it does not overlap any known transcript in truffle v1.0 and its FPKM (Fragments Per Kilobase of exon per Million fragments mapped) value is at least four in the 95% FPKM confidence interval with a class code “u” in the output of Cuffcompare (i.e., unknown, intergenic transcript). Finally, to claim these as novel genes, the overlapping novel transcripts are clustered into a single interval, i.e., the longest transcript, that spans across all the overlapping transcripts. The clusters of overlapping novel transcripts are considered novel genes if their sequences are homologous to any known protein (BlastX analysis via Blast2GO) [23]. After removal of 11 extremely long genes (≥36,573 bp, the longest known gene in truffles, see Additional file 1: Figures S1 and S2 for the length distributions), we identified a total of 614 novel genes, 200 to 500 of which present in each sample (Table 4). A complete list of novel genes along with expression levels and predicted protein functions are available in Additional file 2: Table S1.

**Table 4:** Prediction of genes in RNA-seq datasets

**Sample**	**Reference assembly**	**Number of novel genes**
FB	Truffle v1.0 (7,496 genes)	282
FLM		328
5-aza treated		466
5-aza untreated		440
Merged		614

FB: FruitBody; FLM: Free-Living Mycelium.

### DNA methylation and transcription analysis

**Figure 2:**
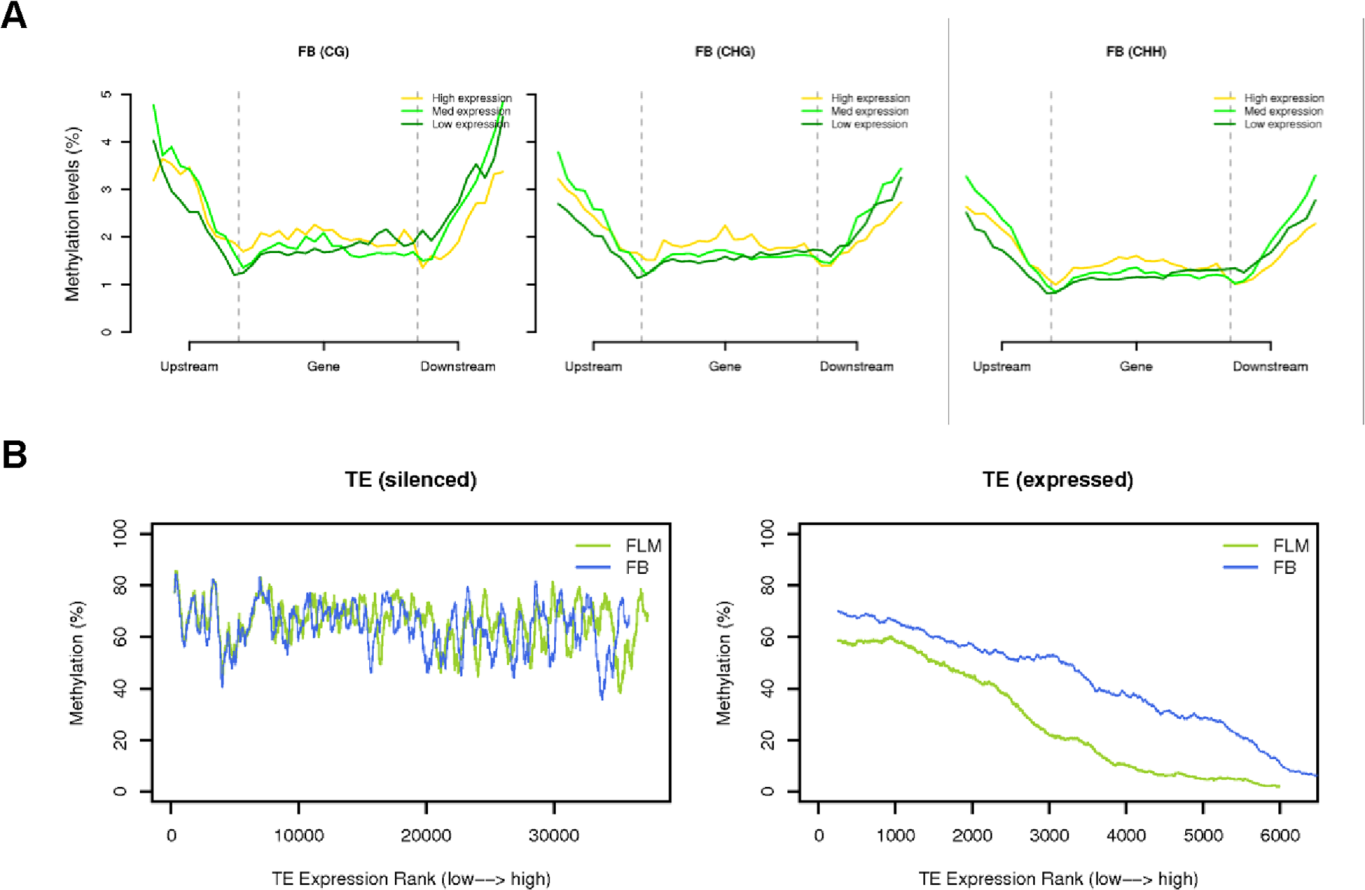
**Integrative analysis of DNA methylation and transcription in genes and transposable elements. A**: Meta-gene plots of CG/CHG/CHH methylation level in high/med/low expression genes. **B**: Methylation level of TEs ranked by the expression level from low to high in silenced (bottom left) and expressed (bottom right) TEs. TE: Transposable elements.

To investigate the relationship between DNA methylation and transcription we performed a joint analysis of the BS-seq and RNA-seq data on genes and TEs. The results highlight a very different pattern between genes and TEs. As shown in Figure 2A, genes are mostly methylation depleted in all contexts, with no clear difference in methylation levels between genes expressed at high, medium or low levels. This argues against a possible involvement of gene body methylation in gene expression regulation. On the other hand, silenced TEs are generally highly (70-80%) methylated, and a clear anti-correlation is apparent between DNA transcription and methylation for the expressed TEs, thus suggesting TE expression regulation by DNA methylation (Figure 2B).

### Transcriptome analysis of truffle mycelia treated with 5-aza

**Figure 3:**
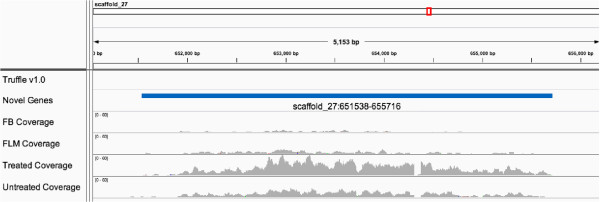
Screenshot of a novel gene identified in 5-aza treated mycelia.

We recently showed that mycelia treated with the demethylating agent 5-aza exhibit a 5-aza concentration-dependent transition to a fluffy-like phenotype [11]. 5-aza treated mycelia also displayed decreased global methylation levels compared to untreated samples. A reactivation of TE transcription was also reported. Here we performed a functional analysis of the genes differentially expressed between untreated and 5-aza treated mycelia using Blast2GO [23]. As revealed by this analysis, untreated vs. 5-aza treated differential transcripts are enriched in genes involved in the oxidation-reduction process and transmembrane transport. The other genes code for transcription factors or for proteins involved in metabolism, response to stimuli, and mycelium development (Table 5). In addition to transcriptional changes, we also identified 68 novel genes/transcripts that are only present in the treated, but not in the untreated sample. Figure 3 shows a novel gene identified in treated, but not in untreated mycelium. Together with our previous findings, this result further supports the existence of an epigenetic regulatory system operating in response to environmental stress exposure in truffles.

**Table 5:** Differentially expressed genes between untreated and 5-aza treated mycelia

Gene*	**Description**	**RPKM No aza**	**RPKM aza**	**Fold Change**	**adj. p value**
***Oxidation-reduction process***					
GSTUMT00004482001	Flavin-binding monooxygenase-like protein	8	258	33.80	3.51E-09
GSTUMT00009102001	Extracellular dioxygenase	18	196	10.71	1.33E-09
GSTUMT00002834001	Alpha-ketoglutarate-dependent taurine dioxygenase	47	414	8.84	4.30E-09
GSTUMT00006862001	Alcohol dehydrogenase 1	4355	34021	7.81	4.81E-08
GSTUMT00005793001	Bilirubin oxidase	1590	8041	5.06	2.28E-05
GSTUMT00000137001	Cytochrome P450	3	17	5.06	5.85E-04
GSTUMT00006911001	Multicopper oxidase	19	90	4.64	4.87E-03
GSTUMT00008228001	Zinc-binding oxidoreductase	34	147	4.35	3.20E-04
GSTUMT00002706001	Alpha-ketoglutarate-dependent sulfonate dioxygenase protein	4	14	3.61	3.51E-02
GSTUMT00000322001	Fatty acid oxygenase	137	48	0.35	4.81E-02
GSTUMT00000528001	Short-chain alcohol dehydrogenases	334	110	0.33	1.22E-02
GSTUMT00007846001	FAD binding domain-containing protein	172	56	0.33	1.54E-02
GSTUMT00002653001	Pyridoxal reductase	104	31	0.30	5.83E-03
GSTUMT00000530001	FAD binding domain-containing protein	61	18	0.30	8.39E-03
GSTUMT00012134001	Dopa -dioxygenase	4704	1368	0.29	3.10E-03
GSTUMT00000158001	Ferritin ribonucleotide reductase-like protein	54	15	0.28	4.52E-03
GSTUMT00002858001	3-oxoacyl-(acyl-carrier-protein) reductase	22	5	0.25	8.53E-03
GSTUMT00012200001	Malate synthase	71	15	0.21	2.41E-03
GSTUMT00001645001	Zinc-binding alcohol	219	34	0.16	1.06E-06
GSTUMT00006980001	Alcohol dehydrogenase	1022	146	0.14	1.36E-07
GSTUMT00007117001	D-isomer specific 2-hydroxyacid dehydrogenase	4	0.4	0.10	4.66E-02
***Transmembrane transport***					
GSTUMT00001488001	Amino acid permease	163	737	4.53	9.07E-03
GSTUMT00003668001	Carboxylic acid transport protein	20	82	4.03	6.04E-03
GSTUMT00010777001	Mate efflux family protein	16	66	4.03	1.22E-02
GSTUMT00008972001	Lactose permease	16	64	4.02	7.12E-03
GSTUMT00001400001	MFS efflux transporter	86	295	3.44	5.58E-03
GSTUMT00000284001	Mg2+ transporter family-like protein	1082	366	0.34	2.56E-02
GSTUMT00008966001	MFS general substrate transporter	27	9	0.34	4.85E-02
GSTUMT00000070001	MFS multidrug transporter	119	30	0.25	1.20E-03
GSTUMT00004586001	ABC transporter	32	5	0.17	3.72E-02
GSTUMT00005001001	MFS drug efflux	663	84	0.13	4.47E-08
***Transcription factors***					
GSTUMT00005836001	Fungal transcriptional regulatory protein	1	18	17.23	1.91E-09
GSTUMT00010369001	STE12-like transcription factor	1	7	12.50	1.93E-04
GSTUMT00004615001	Binuclear zinc transcription factor	2	10	6.40	1.95E-04
GSTUMT00004613001	GAL4 domain-containing protein	90	402	4.49	1.03E-02
GSTUMT00003504001	C2H2 finger domain	1522	467	0.31	4.98E-03
***Metabolic process***					
GSTUMT00008986001	Glycoside hydrolase family 61 protein	0.2	5	25.60	1.10E-04
GSTUMT00001850001	Endo- -beta-glucanase eng1	94	1499	16.02	4.71E-13
GSTUMT00012011001	Glycoside hydrolase family 28 protein	7	57	7.73	3.97E-04
GSTUMT00005539001	Dynamin family protein	27	195	7.18	2.59E-05
GSTUMT00010778001	Agmatinase 1	230	1316	5.72	1.70E-05
GSTUMT00011981001	Acetyltransferase	298	1376	4.62	1.62E-04
GSTUMT00007852001	Alpha amylase	166	704	4.25	6.60E-04
GSTUMT00003032001	Polysaccharide deacetylase	1222	4444	3.64	3.18E-03
GSTUMT00001583001	Pirin	273	968	3.54	3.99E-03
GSTUMT00005439001	Glycerophosphoryl diester phosphodiesterase	214	758	3.54	1.08E-02
GSTUMT00001660001	Glycoside hydrolase family 16 protein	109	374	3.43	6.66E-03
GSTUMT00006417001	Fatty acid activator	417	1410	3.38	5.16E-03
GSTUMT00007213001	Tat pathway signal sequence	74	239	3.25	1.98E-02
GSTUMT00002512001	Gnat family N-acetyltransferase	99	311	3.14	2.83E-02
GSTUMT00000969001	Branched-chain amino acid aminotransferase	840	270	0.32	9.18E-03
***Response to stimuli/mycelium development***					
GSTUMT00004040001	Regulator of G protein signaling domain protein	54	209	3.84	1.40E-03
GSTUMT00007138001	Osmolarity two-component sensor histidine kinase SLN1	401	1134	2.83	4.81E-02
GSTUMT00004012001	Annexin	72	23	0.33	1.38E-02
***Others***					
GSTUMT00004681001	RNase P RPR2 RPP21 subunit domain-containing protein	52	339	6.53	9.70E-07
GSTUMT00002939001	Allergenic cerato-platanin ASP F13	361	1730	4.80	5.46E-05
GSTUMT00010365001	Putative endo-glucanase	8	39	4.66	1.93E-02
GSTUMT00006611001	Protoglobin protein	999	3827	3.83	3.11E-03
GSTUMT00006718001	Glutathione S-transferase	228	80	0.35	3.10E-02
GSTUMT00008071001	XAA-pro dipeptidase	1272	413	0.32	1.27E-02
GSTUMT00001676001	Arrestin (or S-antigen) N-terminal domain protein	96	31	0.32	1.22E-02
GSTUMT00006632001	P-loop containing nucleoside triphosphate hydrolase	3053	724	0.24	2.20E-04

*One hundred fifteen genes undergo a significant change in expression levels (adjusted p value <0.05) upon 5-aza treatment. Functional annotation of the differentially expressed genes/sequences (either up- or down-regulated) was performed with the Blast2GO suite. Fifty-three genes code for hypothetical proteins. The remaining 62 genes were classified into distinct functional categories. “Oxidation-reduction process” and “transmembrane transport” appear as enriched GO terms (Fisher exact test p = 2.6E-4 and p = 7.9E-3, respectively) among differentially expressed genes.

### The composite RIP index (CRI) computation

The composite RIP index (CRI, [12]; see below ‘Availability and requirements of Software Used’) was implemented to measure CA = > TA enrichment within repeated sequences from *Tuber* and other organisms’ genomes. The Composite RIP Index (CRI) was calculated as (RIP product)-(RIP substrate) [12]. The RIP product score $\left(\frac{TpA}{\mathrm{ApT}}\right)$ measures the frequency of RIP products taking into account potential false positives due to local density, while the RIP substrate score $\left(\frac{CpA+TpG}{ApC+GpT}\right)$ measures the depletion of RIP targets and their reverse complement (e.g., CpA, and TpG).

To evaluate and compare CRI values in different organisms, we predicted repeats in *T. melanosporum, Neurospora crassa, Uncinocarpus reesii, Aspergillus nidulans* and *Saccharomyces cerevisiae.* Repeat annotations were predicted from the corresponding genome sequences [24-27] by RepeatMasker [28]. Further details on CRI implementation, including raw data, processed data, scripts (python and R) and output files can be accessed at [29].

## Conclusions

We provide a comprehensive genomic, epigenomic and transcriptomic sequencing data resource for the black truffle *T. melanosporum*. The use of a reversible, rather than an irreversible mechanism (such as RIP) to cope with the multitude of repeated transposable elements that populate the *T. melanosporum* genome resembles the situation in more complex organisms, including humans, and is in line with the view of a “generative” (i.e., genome-shaping) rather than a purely “destructive” role of transposable elements [30]. More targeted follow-up studies built upon the results of the present work may uncover variations of the DNA methylation and transposon activity profiles associated to functionally interpretable (presumably adaptive) modifications of gene expression. If extended to *T. melanosporum* specimens from different geographic areas, epigenomic analyses, such as the one described in this work, may shed light on the relationships between DNA methylation, transposon-mediated genome shaping and commercially relevant organoleptic properties, such as aroma. In addition to their general evolutionary biological implications, our findings may thus provide a new mechanistic layer to explain intraspecific variability.

## Availability and requirements of Software Used

### Implementation of the Composite RIP Index

•**Project name:** The composite RIP index (CRI) computation

•**Project home page:**https://github.com/wwliao/critool/

•**Operating system(s):** Platform-independent

•**Programming language:** Python and R

•**Other requirements:** Python 2.7, R

•**License:** MIT

•**Any restrictions to use by non-academics:** None

## Availability of supporting data

For all sequencing data, in addition to those already described in [11] (GSE49700) we provide several formats to facilitate downstream data analysis: BS-seq data is presented in several formats -- a BAM file of alignments, ATCGmap for SNP calling and coverage analysis, CGmap for methylation analysis and a wiggle file for data visualization on genome browsers, such as IGV [31] (see Additional file 1: Table S1). For WG-seq data, BAM and ATCGmap are provided. For RNA-seq data, we provide BAM files and novel genes detected from the merged transcriptome assembly (see Additional file 2: Table S1). All the datasets presented here are publicly available in the *GigaScience* repository, GigaDB [19].

## Abbreviations

5-aza: 5-azacytidine; 5mC: 5-methylcytosine; CNV: Copy Number Variation; CRI: Composite RIP Index; DMT: DNA Methyl Transferase; ECM: ECtoMycorrhiza; FB: FruitBody; FLM: Free-Living Mycelium; gDNA: genomic DNA; RIP: Repeat-Induced Point mutation; RPKM: Reads Per Kilobase per Million mapped reads; TE: Transposable Element.

## Competing interests

The authors declare that they have no competing interests.

## Authors’ contributions

BM and MM have been in charge of truffle material manipulation, DNA and RNA extraction, and prepared the libraries. BM, PYC and WWL performed BS-sequencing and the bioinformatic analysis. AJ and DL performed RNA-seq analysis. SO and MP planned and supervised the study and wrote the paper. All authors read and approved the final manuscript.

## Supplementary Material

Additional file 1: Figure S1 Histogram of gene length in Truffle v1.0. **Figure S2.** Histogram of gene length of the 614 novel genes. **Table S1.** Data file formats. Click here for file

Additional file 2: Table S1 Novel genes with expression levels and predicted protein functions. Click here for file
