# Supplementary material for: A comprehensive resource of genomic, epigenomic and transcriptomic sequencing data for the black truffle Tuber melanosporum
Source: Gigascience. 2014 Oct 30;3:25. doi: 10.1186/2047-217X-3-25 (PMC4228822; doi:10.1186/2047-217X-3-25)
Supplement: Additional file 1: Figure S1 — Histogram of gene length in Truffle v1.0. Figure S2. Histogram of gene length of the 614 novel genes. Table S1. Data file formats. [file 2047-217X-3-25-S1.docx]

**Supplementary Figure 1**

Histogram of gene length in Truffle v1.0


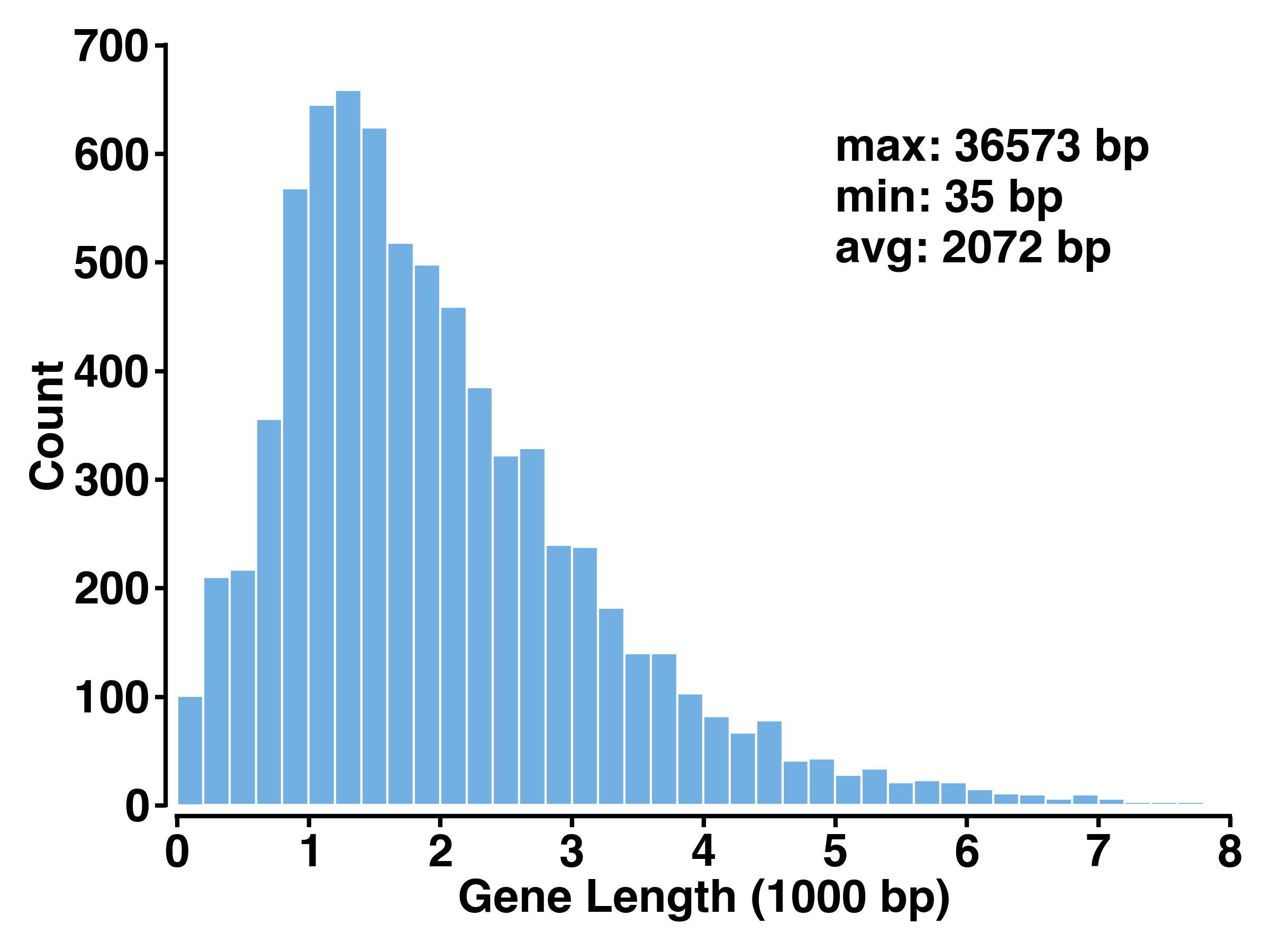


**Supplementary Figure 2**

Histogram of gene length of the 614 novel genes


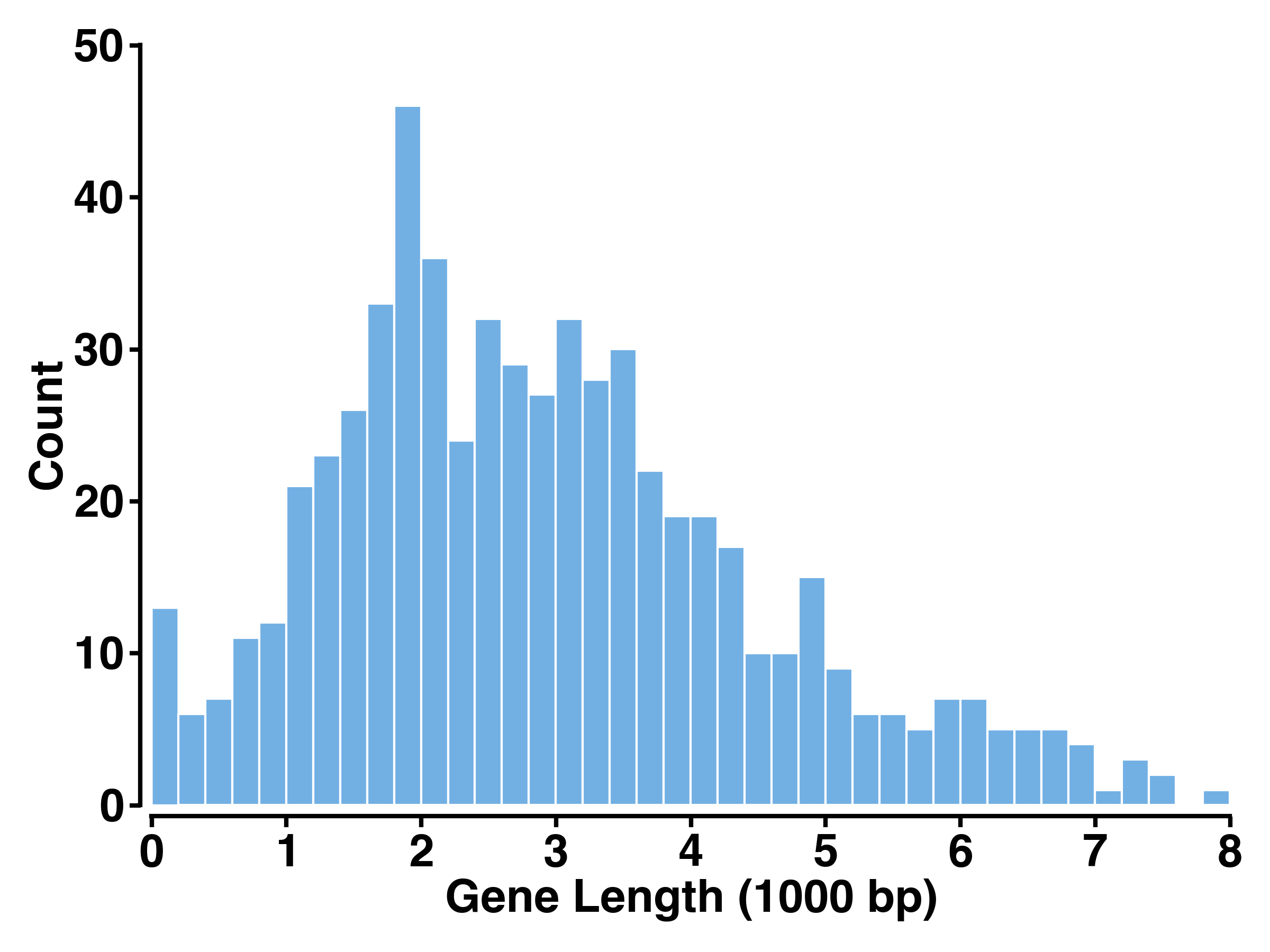


**Supplementary Table 1**

|  | BAM | ATCGmap | CGmap | WIG |
| --- | --- | --- | --- | --- |
| BS-seq | ✓ | ✓ | ✓ | ✓ |
| WG-seq | ✓ | ✓ | N/A | N/A |
| RNA-seq | ✓ | N/A | N/A | N/A |
